# Generation of desminopathy in rats using CRISPR‐Cas9

**DOI:** 10.1002/jcsm.12619

**Published:** 2020-09-07

**Authors:** Henning T. Langer, Agata A. Mossakowski, Brandon J. Willis, Kristin N. Grimsrud, Joshua A. Wood, Kevin C.K. Lloyd, Hermann Zbinden‐Foncea, Keith Baar

**Affiliations:** ^1^ Department of Physiology and Membrane Biology University of California Davis CA USA; ^2^ Department of Neurology with Experimental Neurology Charité – Universitätsmedizin Berlin, Corporate Member of Freie Universität Berlin, Universität zu Berlin, and Berlin Institute of Health Humboldt CA USA; ^3^ Mouse Biology Program University of California Davis CA USA; ^4^ Dept. of Pathology, School of Medicine University of California Davis CA USA; ^5^ Dept. of Surgery, School of Medicine University of California Davis CA USA; ^6^ School of Kinesiology, Faculty of Medicine Universidad Finis Terrae Santiago Chile; ^7^ Neurobiology, Physiology and Behavior University of California Davis CA USA

**Keywords:** Precision medicine, Muscular dystrophy, Injury, Exercise, Force transfer

## Abstract

**Background:**

Desminopathy is a clinically heterogeneous muscle disease caused by over 60 different mutations in desmin. The most common mutation with a clinical phenotype in humans is an exchange of arginine to proline at position 350 of desmin leading to p.R350P. We created the first CRISPR‐Cas9 engineered rat model for a muscle disease by mirroring the R350P mutation in humans.

**Methods:**

Using CRISPR‐Cas9 technology, *Des* c.1045‐1046 (AGG > CCG) was introduced into exon 6 of the rat genome causing p.R349P. The genotype of each animal was confirmed via quantitative PCR. Six male rats with a mutation in desmin (*n* = 6) between the age of 120–150 days and an equal number of wild type littermates (*n* = 6) were used for experiments. Maximal plantar flexion force was measured *in vivo* and combined with the collection of muscle weights, immunoblotting, and histological analysis. In addition to the baseline phenotyping, we performed a synergist ablation study in the same animals.

**Results:**

We found a difference in the number of central nuclei between desmin mutants (1 ± 0.4%) and wild type littermates (0.2 ± 0.1%; *P* < 0.05). While muscle weights did not differ, we found the levels of many structural proteins to be altered in mutant animals. Dystrophin and syntrophin were increased 54% and 45% in desmin mutants, respectively (*P* < 0.05). Dysferlin and Annexin A2, proteins associated with membrane repair, were increased two‐fold and 32%, respectively, in mutants (*P* < 0.05). Synergist ablation caused similar increases in muscle weight between mutant and wild type animals, but changes in fibre diameter revealed that fibre hypertrophy in desmin mutants was hampered compared with wild type animals (*P* < 0.05).

**Conclusions:**

We created a novel animal model for desminopathy that will be a useful tool in furthering our understanding of the disease. While mutant animals at an age corresponding to a preclinical age in humans show no macroscopic differences, microscopic and molecular changes are already present. Future studies should aim to further decipher those biological changes that precede the clinical progression of disease and test therapeutic approaches to delay disease progression.

## Introduction

Desminopathies or desmin‐related myopathies are a clinically heterogeneous group of myofibrillar myopathies caused by mutations of the desmin gene or its interactive partners. To date, more than 60 mutations in the desmin (*DES*) gene have been reported, the majority of which follow an autosomal‐dominant inheritance^1^; however, autosomal‐recessive cases and sporadic patients have been reported.^2^ There seems to be a vague correlation between the genotype and the phenotype^2^; however, symptoms and disease progression vary widely.^3^ Most patients first develop symptoms in their 30s, although the age of onset ranges from infancy to late adulthood.^4^, ^5^, ^6^ Desminopathy is associated with progressive skeletal myopathy and cardiomyopathy. The skeletal muscle myopathies may present as a classic progressive bilateral distal myopathy, but scapuloperoneal, limb girdle, and generalized myopathy phenotypes have also been reported.^7^, ^8^, ^9^ When the diaphragm is affected, respiratory insufficiency can lead to further disability and higher mortality.^10^ *DES* mutations can lead to different types of cardiomyopathy, oftentimes accompanied by cardiac conduction disease.^11^, ^12^, ^13^, ^14^, ^15^ The progressive impairment of muscle function often leads to limitation of mobility, loss of ambulation, and dependence on assistive devices. The result is that life expectancy is reduced, mainly as a result of cardiac and pulmonary complications. No specific treatment for desminopathies exists to date.

The exact molecular pathogenesis of desminopathies remains to be fully understood. Desmin is the primary intermediate filament of striated muscle and forms a three‐dimensional network between the contractile apparatus, the extracellular matrix, and other cell organelles like mitochondria, T‐tubules, and nuclei.^16^, ^17^, ^18^ This continuous cytoskeleton network promotes structural integrity, mechanotransduction, and mechanochemical signalling, making desmin a significant contributor to the maintenance of skeletal muscle structure and function. Disease‐causing mutations are spread over the entire *DES* gene on chromosome 2q35.^1^ Most *DES* mutations generate desmin protein incapable of forming the physiological intracellular filamentous network, causing an accumulation of intracellular aggregates containing desmin and other cytoskeletal proteins.^19^ Misfolded desmin resists degradation and recycling by the cellular enzymatic machinery.^20^, ^21^ Consequently, the main histopathological hallmarks of desminopathy are sarcoplasmic and subsarcolemmal desmin‐positive protein aggregates.^22^, ^23^, ^24^, ^25^ Other myopathic features include increased fibre size variability, central nuclei, atrophic or necrotic fibres, fibre splitting, mitochondrial dysfunction, rimmed vacuoles, inflammation and increased intramuscular connective, and fat tissue.^22^, ^26^, ^27^, ^28^ However, these features vary significantly from patient to patient.

To elucidate the molecular mechanisms in this disease, several animal models have been developed in zebrafish and mice. Although the first mouse *Des* knock‐out models yielded essential insights into desmin function, the total lack of desmin and therefore desmin aggregates was, among others, a major limitation in comparing the model with the human disease.^29^, ^30^, ^31^, ^32^ Since the original knock‐out, various knock‐in and transgenic mouse models have been established and studied. These models, however, continued to have varying success in reflecting the human disease course, as some only developed a mild or even no obvious cardiac phenotype and only homozygous mice showed skeletal myopathy.^20^, ^33^, ^34^, ^35^, ^36^

Here, we present the first rat model of desminopathy created using CRISPR‐Cas9 technology. The model mirrors the R350P *DES* mutation, the most frequent genotype causing desminopathies in humans.^7^, ^8^, ^20^ It is caused by a single amino acid exchange from arginine to proline at position 350, and as rodent desmin lacks a serine at position 82, the ortholog of the human missense mutation is created as an R349P mutation in the rat protein. Unlike similar murine models of this mutation, there are several inherent advantages of using a rat model. Rats share more genetic and physiological traits with humans and are approximately an order of a magnitude larger than their murine counterparts.^37^ Having an animal model that more closely resembles the human phenotype would be an advantage in the pursuit of a better understanding of the disease and therapeutic approaches to ameliorate it. As a first characterization of our model, we report here the clinical, myopathological, functional, and molecular findings in young adult mutant rats (homozygous R349P) compared with wild type littermates.

## Methods

### Animals and ethical approval

All procedures were approved by the Institutional Animal Care and Use Committee of the University of California, Davis, which is an AAALAC‐accredited institution. Animal housing was in accordance with recommendations of the *Guide for the Care and Use of Laboratory Animals*. Animals were housed in a conventional vivarium and were specific pathogen free from the following pathogens: all ectoparasites and endoparasites, sialodacryoadenitis virus (RCV, SDAV), Sendai, Pneumonia Virus of Mice (PVM), Hantavirus, Rat Parvoviruses [RPV, RV (KRV), H‐1], Reovirus (REO‐3), Lymphocytic Choriomeningitis Virus (LCMV), Theiloviruses [MEV (TMEV), RTV (GDVII)], *Cilia‐Associated Respiratory Bacillus* (CAR Bacillus; CARB), and *Mycoplasma arthritidis* and *pulmonis*. Male rats between 120 to 150 days were selected to study functional, histological, and biochemical differences in the phenotype of CRISPR‐Cas9 created knock‐in animals carrying a mutation in desmin (*Des*; *n* = 6) or healthy, wild type littermates (WT; *n* = 8). Animals were housed in 12:12 h light–dark cycles and fed *ad libitum*.

### CRISPR‐Cas9‐mediated knock‐in

#### Design and constructs

**Figure 1:**
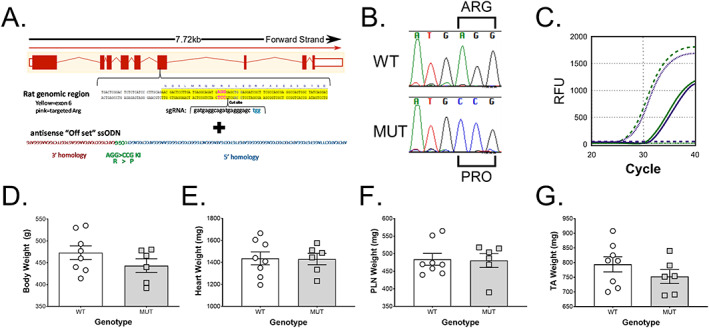
Generation and characterization of CRISPR‐Cas9 engineered rat model of desminopathy. The rat model of R350P desminopathy was created using (A) CRISPR‐Cas9 by engineering the missense mutation, *Des* c.1045‐1046 (AGG > CCG) in exon 6, leading to p.R349P in rats. (B) Sequencing data of wild type (WT) and desmin knock‐in (MUT) rats from the mutation site. (C) The precise two‐point mutation was confirmed by the absence of c.1045‐1046 (AGG > CCG) using specific FAM (green; MUT) or VIC (purple; WT) labelled quantitative PCR probes. WT animals (dotted lines) were VIC^+^ and FAM^−^, MUT animals (dashed line) were FAM^+^ and VIC^−^, and heterozygotic animals (solid lines) were positive for both FAM and VIC. There was no statistical significance (*P* > 0.05) between 120‐day‐old to 150‐day‐old homozygous mutants (MUT, *n* = 6) and their wild type littermates (WT, *n* = 8) regarding (C) bodyweight, (D) heart, (E) plantaris (PLN) or (F) tibialis anterior (TA) muscle weight.

Guide RNA (gRNA) selection targeting rat *Des* utilized the online tool ChopChop (chopchop.cbu.uib.no) to assess optimal gRNA candidates.^38^ The following guide sequence, gatgaggcagatgagggagctgg, was selected and converted into a single guide RNA (sgRNA) using the Precision gRNA Synthesis Kit following manufacturer's instruction (Invitrogen, A29377, Waltham MA). The guide was verified *in vitro* to ensure the ability to completely cleave 400 pmol of PCR fragment produced from the rat genomic region (*Figure* 1A). An ssODN repair template was designed with the engineered Arg to Pro modification approximately one nucleotide (nt) from the Cas9 cleavage site and utilized an offset homology arm method consisting of a 36 nt 3′ arm and 91 nt 5′ arm.^39^ The ssODN was synthesized as an Ultramer (Integrated DNA Technologies, Coralville IA).

### Delivery of Cas9 reagents to rat zygotes

Five‐week‐old Sprague–Dawley rats (Envigo, SD, Livermore CA) were superovulated by sequential intraperitoneal injection of 20 International Units of Pregnant Mare Serum Gonadotropin (Lee BioSolutions, Maryland Heights, MO) followed 47 h later with 25 International Units of human chorionic gonadotropin (Sigma, St. Louis, MO) and then paired overnight with 10‐week‐old male Sprague–Dawley studs. The next morning, female rats with visible copulation plugs were euthanized, and their oviducts collected in M2 media (Zenith, ZFM2‐100, Guilford CT). Clutches of zygotes were released from the ampulla and treated with hyaluronidase (300 U/mL) (Sigma, H4272, St. Louis, MO) in M2 media at room temperature for 3–5 min to remove cumulus cells, washed through three drops of 200 μL KSOMaa media (Zenith, ZEKS‐050) and cultured with 200 μL drops of KSOMaa media in an incubator at 37°C and 5% CO_2_ until either microinjection or electroporation.

### Microinjection

On the morning of injection, Cas9 protein (Integrated DNA technologies, 1081058, Coralville IA) and the sgRNA were complexed at 37°C for 10 min in 10 μL of 10 mM Tris; 0.1 mM EDTA injection buffer. The ssODN was added and brought up to a volume of 50 μL with final concentrations of 0.13 μM Cas9, 58 μM sgRNA, and 1.2 μM ssODN. The mix was centrifuged at 12 000 rpm for 3 min and 40 μL taken from the top for the injection. The prepared CRISPR/Cas9 solution was backloaded with a microloader into a newly prepared microinjection pipette using a Sutter Instruments Co. P‐97 micropipette puller from a glass capillary tube and set up onto a Nikon micromanipulator microscope system. Injections were performed under 400× magnification using continuous flow positive pressure of 0.1 psi from a Narishige IM‐300 microinjector. A group of up to 100 zygotes with two visible pronuclei was injected under continuous flow with ~2 pL, or until 50% swelling of the pronuclear cavity was observed. The continuous flow also resulted in ~1 pL of reagent being introduced to the cytoplasm. Zygotes were returned to a culture dish of 200 μL drops of KSOMaa in the incubator until surgical transfer to pseudopregnant recipients the same day.

### Electroporation

On the morning of electroporation, a final electroporation mix of 8 μM Cas9 protein (Integrated DNA technologies, 1081058, Coralville IA), 12 μM sgRNA, and 19 μM ssODN was prepared following the previously published electroporation (EZ) method.^40^ Prior to electroporation, a group of up to 40 zygotes were treated with Acidic Tyrode's solution (Sigma, T1788, St. Louis MO) for ~30 s or until ~30% zona erosion was observed. The zygotes were then immediately washed through four 50 μL drops of M2 media with 1% bovine serum albumin (Sigma, A3311, St Louis, MO) then combined with the ribonucleoprotein mixture in a 20 μL final volume mixture. The zygotes with then electroporated with six pulses each lasting 3 ms in duration with 100 ms between pulses at 30 V using a Bio‐Rad Gene Pulser XCell (Bio‐Rad, Hercules, CA) in a 0.1 cm cuvette (Bio‐Rad, #1652089,Hercules, CA). The zygotes were then recovered and cultured in 200 μL drops of KSOMaa in the incubator until surgical transfer to pseudopregnant recipients the same day.

### Zygote transfer

Synchronization of the 9‐week‐old Sprague–Dawley recipient females was achieved using injection of 40 μL IP injection of luteinizing hormone releasing hormone agonist (Sigma Aldrick Inc, L4513‐5MG, Milwaukee WI) then paired overnight with 10‐month‐old vasectomized males 24 h prior to zygote transfer surgery to induce pseudopregnancy. The next day, female rats with visible copulation plugs were anaesthetized with isoflurane (Fluriso, VetOne, Boise ID) (2–5%) for the embryo transfer surgical procedure. After sterile preparation of the surgical site, a dorsal midline incision through the skin was performed. An incision was made through the body wall to exteriorize the oviduct, and a small opening was made with a 26‐gauge needle near the infundibulum in order to transfer approximately 10 zygotes into the ampulla. This procedure was then repeated for the opposite oviduct. After transfer, the oviducts were returned to the abdominal cavity, the muscle wall was sutured closed, and the skin was closed using surgical staples. Upon completion of the surgery, animals received 0.1 mg/kg Buprenorphine (JHP Pharmaceuticals, Parsippany, NJ) (0.03 mg/mL). Recipients were placed on heating pads and had respiration rate and anaesthesia recovery closely monitored until becoming fully ambulatory.

### Genetic screening

Genomic DNA was extracted from approximately 3 mm tail snips utilizing DNeasy Blood & Tissue Kit following manufacturer's instruction (Qiagen, 69504, Hilden Germany). Approximately 50 ng of DNA was PCR amplified with a gene specific forward primer, CCAGATGAGTGGATTCCTAGTTGGG, and reverse primer, CACCTAAGCTCTCACCTGCTCTCCT, producing 546 bp amplicon. Fifteen microlitres PCR reactions included 0.4 μM of each primer, 1X PCR buffer, 1.7 mM MgCl2, 0.2 mM each dNTPs, 1 Unit Amplitaq polymerase (Applied Biosystem, N8080153, Waltham MA), and 1.3 M Betaine, 1.3% DMSO (Sigma, St. Louis, MO). Thermal cycling included an initial denaturing at 94°C for 5 min; 10 cycles of 94°C for 15 s, 65°C to 55°C for 30 s (↓1°C/cycle), 72°C for 40 s; 30 cycles of 94°C for 15 s, 55°C for 30 s, 72°C for 40 s; final extension of 72°C for 5 min and maintained at 4°C. PCR reactions included a non‐template control, negative wild type control (B6). Subsequent amplicons were separated on 1% Agarose gel for 60 min at 120 V, and appropriately sized amplicons were gel extracted using the QIAquick Gel Extraction Kit following manufacturer's instruction (Qiagen, 28115, Hilden Germany). Purified fragments were submitted for Sanger sequencing and analysed using Sequencher sequence analysis software (Gene Codes, Ann Arbor MI). For screening, the amplicon was quantified using probes that recognized either the WT, tagged with VIC, or MUT sequence, coupled to the FAM fluorophore (*Figure* 1B).

### Surgical intervention and muscle collection

#### Synergist ablation surgery

Rats were anaesthetized using 2.5% inhaled isoflurane vaporized in oxygen. The right gastrocnemius and soleus muscles were isolated and excised at the Achilles tendon while leaving the plantaris (PLN) muscle, blood, and nerve supply intact. The overlying fascia and skin were closed separately using sterile suture and clips, respectively. All animals were treated with analgesics to ease post‐operative pain (buprenorphine, 0.1 mg kg^−1^) and monitored daily for signs of pain or post‐operative infection.

### Muscle collection

Control and synergist ablated (14 days postoperative) rats were anaesthetized with 2.5% inhaled isoflurane, the PLN was excised from both hindlimbs, rinsed in phosphate buffered saline to remove blood, blotted dry, and weighed. Subsequently, the PLN was pinned on cork at resting length and frozen in liquid nitrogen‐cooled isopentane for histological and biochemical analyses. Tibialis anterior muscles, heart, liver, and fat were also collected, blotted dry, and weighed before freezing at −80°C.

### Histology

The frozen PLN muscles were blocked, and serial cross‐sections (10 μm) were cut from the PLN using a Leica CM 3050S cryostat (Leica Microsystems, Buffalo Grove, IL, USA). For haematoxylin and eosin (H&E) staining, sections were brought to room temperature, then consecutively submerged in EtOH 90% and then tap water for 30 s. Four hundred microlitres Mayer's hemalum solution (Merck, Darmstadt, Germany) were applied per slide and left to incubate for 6 min. Slides were then blued under running lukewarm tap water for 2 min, then dipped in tap water for another 8 min. Three hundred microlitres 0.5% aqueous eosin γ‐solution (Merck, Darmstadt, Germany) were applied to each slide for 1 min. Sections were dehydrated in sequential steps using 70%, 90%, and 100% EtOH for 1 min each. Alcohol was removed by submerging sections in Xylene for 5 min. After sections were air dried, they were mounted with DPX Mountant for histology (Sigma, Darmstadt, Germany).

For immunohistochemistry, PLN muscle sections were fixed in cold acetone for 5 min at −20°C, followed by three 5 minute phosphate‐buffered saline washes in 0.1% Tween‐20 before blocking with 5% natural goat serum (NGS) for 30 min at room temperature. Sections were then incubated with primary antibody overnight at 4°C. For determination of fibre types, SC‐71 (myosin heavy chain 2A, mouse, IgG1, 1:250 in NGS), BF‐F3 (myosin heavy chain 2B, mouse, IgM, 1:250 in NGS), and a polyclonal laminin antibody (rabbit, IgG (H + L), 1:500 in NGS) were used. For detection of desmin aggregates, a monoclonal desmin antibody (mouse, IgG2a, 1:200 in NGS) was used. After incubation in primary antibody, sections were washed three times with phosphate‐buffered saline washes in 0.1% Tween‐20 for 5 min and then incubated in secondary antibody (goat anti‐mouse Alexa Fluor® 488 and 555, Life Technologies and goat‐anti‐rabbit AlexaFluor® 647 for fibre typing, goat anti‐mouse Alexa Fluor® 488 for desmin) for 30 min at room temperature. Sections were mounted using ProLong Gold Antifade reagent (Life Technologies). Anti‐myosin heavy chain antibodies were purchased from the Developmental Studies Hybridoma Bank (Iowa City, Iowa), anti‐laminin antibody was purchased from Sigma Aldrich (St Louis, Missouri), and anti‐desmin antibody was purchased from Santa Cruz Biotechnology (Dallas, Texas).

Slides were imaged using a Leica DMi8 inverted microscope using the HC PL FLUOTAR 10x/0.32 PH1 objective (Leica Microsystems, Wetzlar, Germany) and using the LAS X software. For comparative analysis, exposure length remained fixed for all samples. Whole‐muscle sections were imaged using auto‐stitching with a 10% overlap.

Muscle fibre properties and fibre types were analysed using FIJI and SMASH (MATLAB) software from the whole muscle sections. In FIJI, images were cropped, and all areas with obviously false positive fluorescence, that is debris or folds, were deleted. In SMASH, Initial Segmentation was performed with segmentation factor 5 and appropriate pixel size (1.29 μm). Segmentation was checked and manually corrected, if necessary. The fibre filter parameters used were Minimum Fibre Area 500 μm^2^, Maximum Fibre Area 10 000 μm^2^, Maximum eccentricity 0.95, and Minimum Convexity 0.8. The fibre filter was checked, and false positives (e.g. vessels) were manually deleted. Threshold for fibre typing parameters was assessed and adjusted individually for each image.

Nuclei were analysed from H&E‐stained whole muscle sections using FIJI software with the grid tool. All fibres with distinctly central nuclei were counted except fibres in the two outer fibre layers because fibres at the edge of all sections showed more artefacts. The data were expressed as the percentage of fibres with centralized nuclei.

### Immunoblotting

Frozen muscle trimmings, acquired during sectioning of the PLN for histological analysis, were used for immunoblotting. Frozen PLN muscle sections were homogenized in 200 μL sucrose lysis buffer (50 mM Tris pH 7.5, 250 mM sucrose, 1 mM EDTA, 1 mM EGTA, 1% Triton X‐100, 1% protease inhibitor complex) on a vortexer for 60 min at 4°C. Following centrifugation at 10 000 *g* for 10 min, the supernatant was collected. Protein concentrations were determined in triplicates using the DC protein assay (Bio‐Rad, Hercules, CA, USA). Samples concentrations were adjusted using sucrose lysis buffer, and following dilution Laemmli sample buffer, 1 μg protein/μL were denatured at 100°C for 5 min. Proteins (10–15 μg protein per lane) were loaded on 4–20% Criterion TGX Stain‐free gels (Bio‐Rad) and run for 45 min at 200 V, and the proteins were visualized after a UV‐induced 1 min reaction to produce fluorescence. Following quantification of total protein, proteins were transferred to nitrocellulose membrane at 100 V for 30–60 min, depending on the size of the protein of interest. Efficient transfer was confirmed used Ponceau staining of the membrane. Membranes were then washed and blocked in 1% fish skin gelatin dissolved in Tris‐buffered saline with 0.1% Tween‐20 for 1 h. Membranes were then rinsed and probed with primary antibody overnight at 4°C. The next day, membranes were washed and incubated with HRP‐conjugated secondary antibodies at 1:7500 to 1:10 000 in 1% skim milk‐Tris‐buffered saline with 0.1% Tween‐20 for 1 h at room temperature. Immobilon Western Chemiluminescent HRP substrate (Millipore, Hayward, CA, USA) was then applied to the membranes for protein visualization by chemiluminescence. Image acquisition and band quantification were performed using the ChemiDoc MP System and Image Lab 5.0 software (Bio‐Rad). Protein levels of each sample were calculated as band intensities relative to total protein. The following antibodies were used in this study at a concentration of 1:1000: dystrophin (Santa Cruz, cat. no. 365954, lot no. E2711), *β*‐dystroglycan (Hybridoma Bank, cat. no. MANDAG2 (7D11)), desmin (Santa Cruz, cat. no. 271677, lot no. F1913), desmuslin (Santa Cruz, cat. no. 374484, lot no. B0207), dysferlin (Santa Cruz, cat. no. 16635, lot no. H162), Annexin A2 (Cell Signalling, cat. no. 8235S, lot no. 2), and muscle LIM protein (Santa Cruz, cat. no. 166930, lot no. E2814). Sarcospan and syntrophin cross‐reacted with the antibody for dystrophin (Santa Cruz, Cat. no. 365954) and were determined by molecular weight.^41^

### Statistics

Depending on the number of the groups compared, an unpaired *t*‐test or a two‐way analysis of variance (ANOVA) with a post hoc Tukey's multiple comparisons test was used to test the null hypothesis. An alpha of *P* < 0.05 was deemed statistically significant, and a *P* value between 0.05 and 0.1 was called a trend. Data in the text are reported as mean ± standard deviation, and data in the figures are visually represented as scatter dot plot with error bars indicating standard error of the mean. All analysis was performed with GraphPad Prism Version 7 (La Jolla, CA, USA).

## Results

### Generation of a CRISPR‐Cas9‐engineered rat model for desminopathy

Desminopathy caused by a single nucleotide polymorphism in the *DES* gene that converts arginine 350 to proline (R350P), the most prevalent mutation of desmin in humans, commonly presents as progressive muscular atrophy and muscle weakness. We created an analogue rat model by engineering the rat missense mutation *Des* c.1045‐1046 (AGG > CCG) in exon 6 using CRISPR‐Cas9 leading to p.R349P in rats (*Figure* 1A). Because the length of desmin in the rat is 469 amino acids compared with 470 in humans, R349P in rats is orthologous to R350P in humans. All embryo transfer recipients (*n* = 5) successfully produced litters, and all five recipients implanted with either EZ, microinjection, or a mix of both EZ and microinjection embryos yielded homology directed repair (HDR) positive or potential frameshift insertion or deletion positive offspring (confidence probability of pot + = 0.015). The Precise two‐point mutation was confirmed by the absence of c.1045‐1046 (AGG > CCG) via quantitative PCR (*Figure* 1B) in all HDR offspring. Homozygous rats carrying the mutation in desmin are called MUT throughout the manuscript. The bodyweight of male 120‐day‐old to 150‐day‐old MUT (*n* = 6) was on average 7% lower than wild type littermates (WT) (*n* = 8) without reaching statistical significance [473 ± 45 g (WT) to 443 ± 38 g (MUT)] (*Figure* 1C). Heart weight did not differ between WT and MUT at that age [1437 ± 166 mg (WT) to 1430 ± 127 mg (MUT)] (*Figure* 1D). Neither plantaris [484 ± 48 mg (WT) to 481 ± 48 (MUT)] (*Figure* 1E) nor tibialis anterior muscle weight [794 ± 73 mg (WT) to 753 ± 58 mg (MUT)] (*Figure* 1F) differed significantly either.

### Histological symptoms precede a clinical phenotype

**Figure 2:**
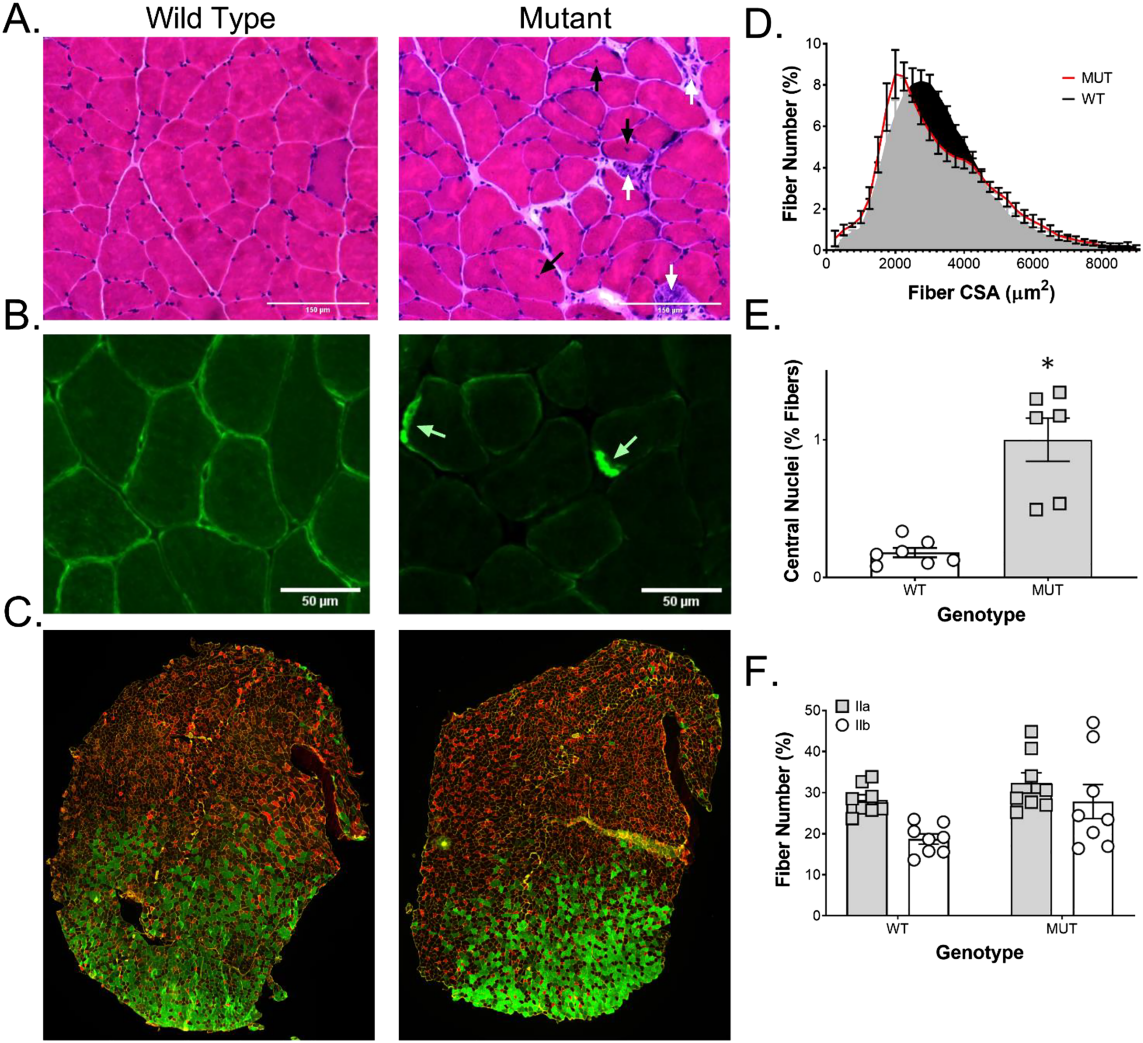
Histological alterations in muscle with MUT desmin protein. H&E staining of plantaris muscle sections showed an (A) increased number of necrotic and regenerating fibres (white arrows) as well as increased number of fibres with central nuclei (black arrows) in desmin mutants. Immunohistochemical staining for (B) desmin (green) showed distribution predominantly around the sarcolemma of WT fibres, but only aggregates (white arrows) in MUT animals. (C) Muscle fibre type analysis showed (F) a tendency for fewer type 2b fibres (green) compared with 2a fibres (red) in WT (*P* = 0.08) and MUT animals (*P* = 0.11). (D) The cross‐sectional area (CSA) calculated from all fibres of plantaris muscle cross sections showed a shift to a higher number of smaller fibres (2000 μm^2^) in mutants (MUT, *n* = 6) compared with wild type littermates (WT, *n* = 8), where the median fibre is substantially larger in the WT (2750 μm^2^). (E) Quantification of central nuclei from H&E‐stained plantaris muscle cross sections showed a significant increase in fibres with central nuclei in mutants (MUT, *n* = 6) compared with wild type littermates (WT, *n* = 7). ^*^ *P* < 0.05.

Cross‐sectional analysis of plantaris muscle sections of WT and MUT stained with H&E revealed early symptoms of myopathy such as increased variation of myofiber size (left shift in median fibre size with a right side shoulder indicating larger fibres), an increased number of regenerating and necrotic fibres, as well as increased number of fibres with central nuclei (*Figure* 2A and 2E). Immunohistochemical analysis of desmin showed an even distribution of desmin around the sarcolemma of WT fibres, but an aggregated distribution of desmin in MUT animals, a classical feature of desminopathy in patients and other animal models (*Figure* 2B).^1^, ^9^, ^20^ Whole muscle cross‐sectional analysis (*Figure* 2C) shows a shift to a higher number of smaller fibres (2000 μm^2^) in MUT compared with WT rats where the highest frequency of occurring fibres is substantially larger (2750 μm^2^) (*Figure* 2D). Because the weight of the MUT plantaris muscle are not different (*Figure* 1E), this would suggest a higher number of fibres in MUT compared with WT. Fibre number in WT tended indeed to be lower than in MUT, albeit not statistically significant [4212 ± 444 (WT) to 4497 ± 271 (MUT)] (data not shown). Muscle fibre type analysis showed a tendency for fewer type 2b fibres compared with 2a fibres in WT (*P* = 0.08) and MUT animals (*P* = 0.11) (Figure 2(F)). No type 1 fibres were found in either WT or MUT, indicating that the remaining fibres in both genotypes express predominantly 2x myosin heavy chain.

### Proteins associated with structural integrity, force transfer, and injury

**Figure 3:**
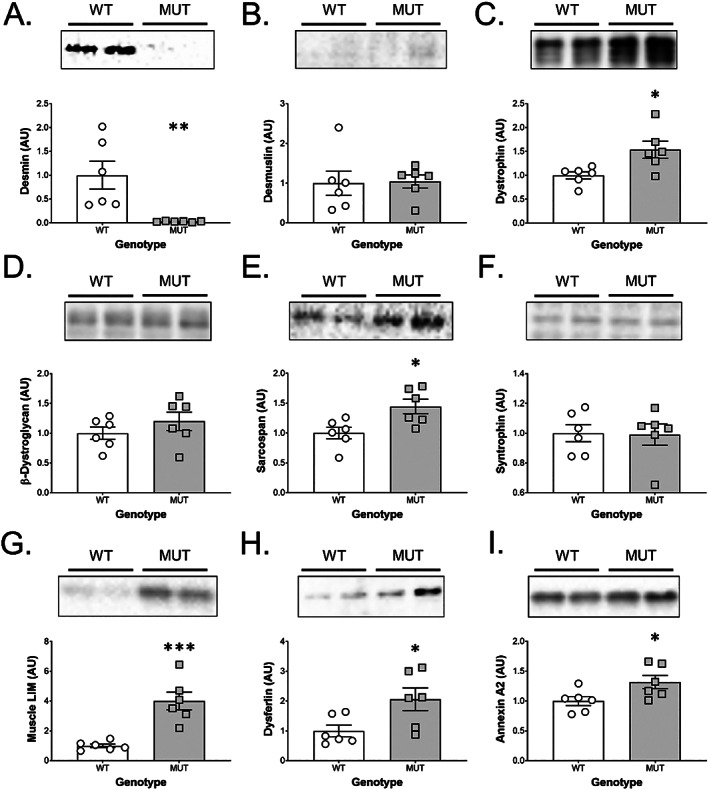
Changes to proteins involved in structural integrity, force transfer and injury in desmin MUT rats. Immunoblotting for proteins important for skeletal muscle structure, force transfer, sarcolemma stability, and indicators of injury revealed several differences between mutant rats (MUT, *n* = 6) and wild type littermates (WT, *n* = 6). (A) Desmin protein levels were 36‐fold lower in MUT compared with WT. (B) Desmuslin, (D) beta‐dystroglycan, and (F) syntrophin did not differ between WT and MUT animals, but (C) dystrophin and (E) sarcospan levels were increased by 54% and 45% in MUT compared with WT animals, respectively. (G) Muscle LIM protein levels were increased by four‐fold in MUT animals compared with WT animals. The membrane repair proteins (H) dysferlin and (I) Annexin A2 were also significantly increased in MUT compared with WT animals. ^*^ *P* < 0.05, ^**^ *P* < 0.01, and ^***^ *P* < 0.001.

We next investigated levels of proteins important for skeletal muscle structure, force transfer, sarcolemma stability, and indicators of injury in MUT muscle compared with wild type littermates (WT). Multiple proteins appeared differentially regulated in MUT compared with WT rats. The MUT animals had 36‐fold lower desmin levels compared with WT (*P* < 0.01) (*Figure* 3A). To investigate potential compensatory effects in MUT animals, we chose to examine proteins known to interact with desmin or to be otherwise involved in the structural integrity of skeletal muscle. Desmuslin (synemin), known to interact with desmin and the dystrophin‐glycoprotein complex, did not differ between WT and MUT animals (*Figure* 3B). However, dystrophin levels were increased by 54% in MUT compared with WT animals (*P* < 0.05; *Figure* 3C). While *β*‐dystroglycan levels did not differ between WT or MUT animals (*Figure* 3D), sarcospan showed a 45% increase in MUT animals compared with WT animals (*P* < 0.05; *Figure* 3E). Syntrophin levels were not different between WT and MUT animals (*Figure* 3F). We found a four‐fold increase in muscle LIM protein levels (CSRP3), a protein known to have a central role in architectural maintenance, in MUT animals compared with WT (*P* < 0.001; *Figure* 3G). Dysferlin, which has been shown to be indispensable in membrane repair and fusion of repair vesicles to the plasma membrane, was increased by two‐fold in MUT compared with WT animals (*P* < 0.05; *Figure* 3H). Annexin A2, which interacts with dysferlin during membrane repair, was also increased in MUT compared with WT animals (*P* < 0.05; *Figure* 3I).

### Overload‐induced injury, muscle growth, and force production

**Figure 4:**
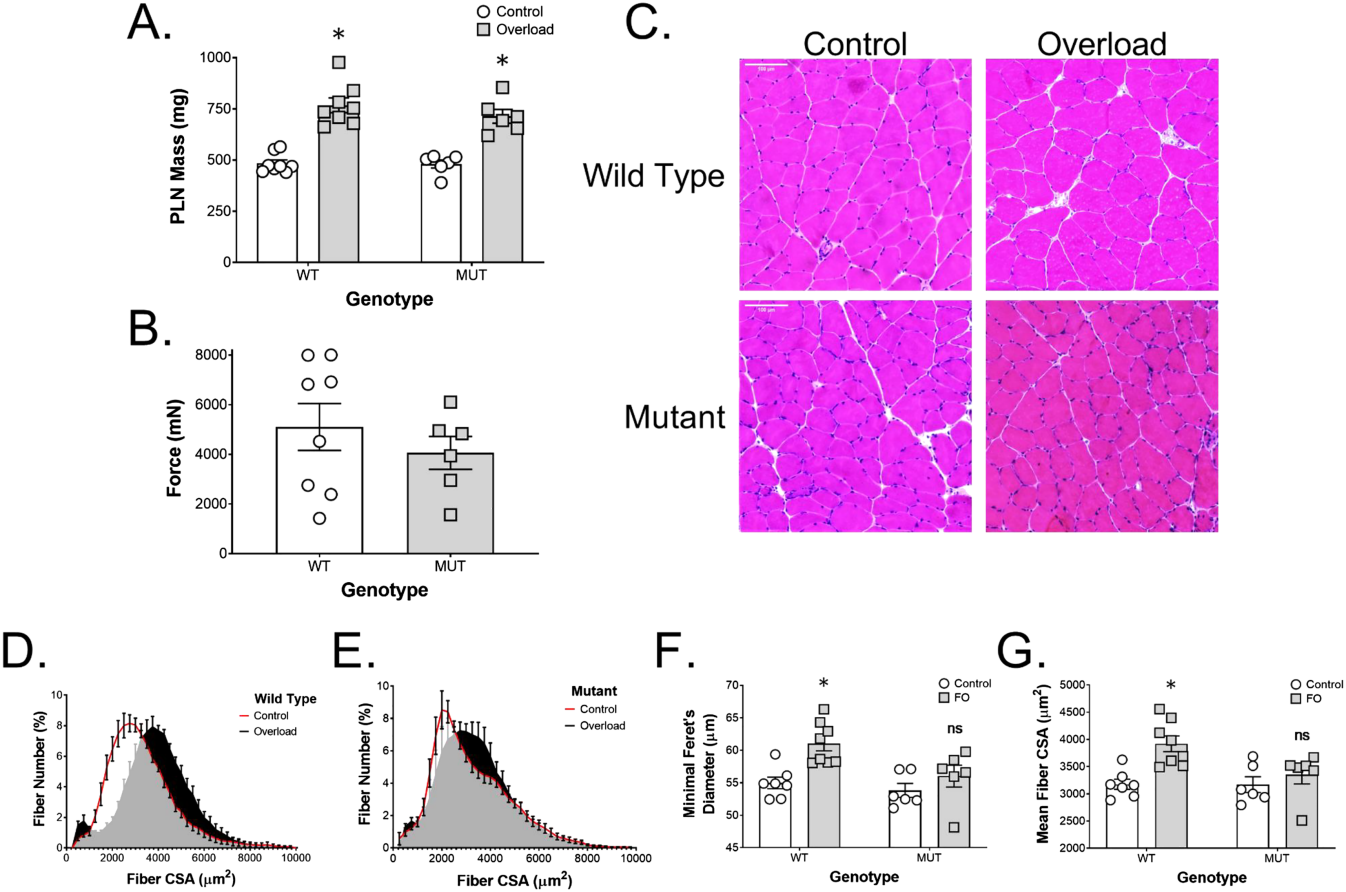
Impaired response to functional overload in desmin MUT rats. Functional overload by ablation of the gastrocnemius and soleus muscles led to an (A) increase in plantaris (PLN) muscle mass of 59% in wild type rats (WT, *n* = 8) and 48% in mutants (MUT, *n* = 6) compared with the non‐ablated control legs. (B) Maximal force of the overloaded plantaris of WT animals was 26% higher than in overloaded MUT animals without reaching statistical significance. (C) H&E staining of plantaris muscle cross sections revealed an increase in fibre size in overloaded WT muscle, but not in overloaded MUT muscle. (D) The cross‐sectional area (CSA) calculated from whole‐muscle cross sections plantaris muscle stained for laminin showed a right shift in WT animals with overload and a distinct increase in very small fibres around 500 μm^2^. (E) MUT animals show a less pronounced right shift of fibre CSA and a smaller increase in small fibres than in WT with overload. Functional overload of plantaris muscle lead to a significant increase in (F) minimum Feret's diameter and (G) mean fibre CSA in WT, but not in MUT animals. ^*^ *P* < 0.05.

To investigate how MUT muscle would respond to a loading challenge, we performed synergist ablation surgery. The gastrocnemius and soleus muscles were removed from the right leg, while the left leg served as an internal control. Synergist ablation is commonly associated with rapid changes in muscle mass and fibre cross‐sectional area.^42^ Indeed, both WT and MUT plantaris muscles responded with stark increases in muscle mass (*Figure* 4A). Plantaris weight increased from 484 ± 48 mg to 767 ± 102 mg in WT animals while it increased from 481 ± 48 mg to 714 ± 82 mg in MUT animals (*Figure* 4A). This is equivalent to a 59% increase in muscle mass in WT rats compared with a 48% in MUT rats. We measured maximal force of the overloaded plantaris of WT animals (5103 ± 2667 nm), which was 26% higher than in overloaded MUT animals (4060 ± 1615 nm) albeit not statistically significantly different based on high variability (*Figure* 4B). In order to investigate desmin MUT on the fibre level, we employed H&E staining (*Figure* 4C) and immunohistochemistry with subsequent quantification of fibre size and distribution, revealing pronounced differences in the ability of MUT fibres to adapt to synergist ablation compared with WT. We found a clear and evenly distributed right shift in fibre CSA in WT animals with overload, the median fibre area shifting from 2750 to 3750 μm^2^ (*Figure* 4D). In addition, there was a distinct increase in very small fibres ~500 μm^2^ in WT animals, potentially indicating new fibres, fibre splitting, or fibre death. In contrast, MUT animals show a less evenly distributed curve under control conditions with the highest frequency of fibres occurring at 2000 μm^2^, which is slightly evened out through overload where the highest frequency of fibres in MUT now matches the baseline condition of healthy littermates at 2750 μm^2^ (*Figure* 4E). The increase in small fibres seen in WT with overload is substantially less pronounced after overload in MUT. These changes in fibre size distribution and ability to adapt to increased loading of skeletal muscle are confirmed by differences in the amplitude of change in minimum Feret's diameter and mean fibre cross sectional area between WT and MUT (*Figure* 4F and 4G). Two‐way ANOVA revealed an effect for genotype (*P* = 0.02) and overload (*P* = 0.002), as well as a trend towards an interaction between genotype and overload (*P* = 0.1) in respect to minimum Feret's diameter (*Figure* 4F). In WT, minimum Feret's diameter increased with overload (*P* < 0.01; *Figure* 4F). In contrast, the increase in Feret's diameter with overload in MUT was not statistically significant (*Figure* 4F). Similarly, two‐way ANOVA of mean fibre cross‐sectional area showed an effect for overload (*P* < 0.01) as well as a tendency for an effect for genotype (*P* = 0.054) and an interaction between overload and genotype (*P* = 0.059; *Figure* 4G). Post hoc analysis showed a significant change in mean fibre cross‐sectional area in WT (*P* < 0.01), but not MUT (*Figure* 4G).

## Discussion

To our knowledge, we have created the first CRISPR‐Cas9‐engineered rat to mimic a human muscle disease. This model mimics early desminopathic changes and might be a good model to investigate interventions to decrease the impact of this devastating disease.

We successfully replicated the exact two‐point mutation in the rat desmin gene, originally identified in a family of patients, using a precision dynamic nuclear polarization knock‐in by single stranded DNA assisted HDR. The result was that a whole body *Des* c.1047 (AGG > CCG) mutation in exon 6 was produced via CRISPR‐Cas9. Both electroporation and microinjection are capable of producing viable HDR offspring following embryo transfer and HDR founders were capable of passing the same 2‐point mutation to their offspring and establish a line of HDR knock‐in rats.

With a mean age of the onset of the first neuromuscular symptoms of 35 years, patients suffering from a mutation in desmin often present fairly late in life compared to other inheritable neuromuscular diseases.^3^ When patients first present, they already suffer from a clinically manifest myopathy. As there is currently no treatment available, damage to the muscle is irreversible, and therapy is usually focused on controlling the symptoms. Therefore, having an animal model that allows researchers to identify early signs of the disease and the underlying biological changes driving the progression of the disease is particularly important to pave the way for interventions that slow or treat the disorder. While there are a number of murine models of desmin mutations available,^20^, ^33^, ^34^ no rat model for desminopathy had been developed. Because rat muscle is an order of magnitude bigger and better mimics the physiology of human muscle,^37^ we felt that a rat model would provide more tissue and better insight into the disease.

In order to be able to investigate early biological changes that precede the strong phenotype at later stages, we chose adult animals between 120 and 150 days of age. This age corresponds to an age in humans of approximately 15–25 years, where most patients with a *DES* R350P mutation have yet to develop severe clinical symptoms.^43^ As in humans, at this age body, heart, and muscle weights were not statistically different. This supports our hypothesis that the age of the animals corresponds to a pre‐clinical stage. In spite of this being a pre‐clinical time point, we found macroscopic, phenotypic, and physiologic differences between MUT and WT animals.

Desmin is an intermediate filament that interacts with microfilaments, other intermediate filaments and microtubules to maintain the structural integrity of the cytoskeleton in cardiac, skeletal, and smooth muscle.^17^, ^44^ Together with syncoilin, it is thought to link the intermediate filaments with the dystrophin‐associated protein complex.^45^ Thus, desmin is not only the main protein responsible for linking adjacent myofibrils to each other but also impacts the connection between the cytoskeleton and the extracellular matrix. Desminopathies are clinically defined as a myofibrillar myopathy characterized by desmin positive protein aggregates and degenerative changes to contractile fibres.^1^, ^46^ These pathological aggregates have been implied to be directly involved in muscle cell degeneration and impaired degradation, potentially via increased oxidative damage.^46^, ^47^ Histochemistry and immunohistochemistry of MUT rats confirmed that these degenerative changes occur before onset of clinical symptoms. Concomitant with aggregate formation, there was an increased number of central nuclei in MUT compared with WT, indicating that an increased number of fibres were undergoing pathological changes. Indeed, central nuclei are a common feature observed in many desminopathies and several other types of myopathies and are thought to be a sign of ongoing myofiber repair.^48^, ^49^, ^50^ In addition to those classic symptoms of desminopathy, we found a left shift towards a higher number of small fibres in MUT compared with WT indicating difficulty to grow to or maintain regular muscle fibre size. In line with this observation, we found a tendency towards more type 2a and 2b fibres and fewer type 2x fibres in MUT compared with WT. Because type 2x fibres together with type 2b fibre are commonly among the larger fibres in rat skeletal muscle and we found a slightly higher number of total fibres in MUT (data not shown), this could explain the fact that the left shift towards smaller fibres was not reflected in decreased muscle weight.

Even though desmin is known to be a major structural protein in skeletal muscle and heart, relatively little is known on the effects of mutated desmin on other structural proteins. We investigated several proteins associated with force transmission, structural integrity, and injury of skeletal muscle. Desmin protein levels were 36‐fold lower in MUT animals than in WT. This is in line with data from Des R349P knock‐in mice, which showed that homozygous animals have almost no desmin when probed for with a regular desmin antibody detecting R349, while they have a distinct band for mutated desmin (P349).^20^ We found that with this decrease in functional desmin protein, many other major structural protein levels were increased. Dystrophin levels were 54% higher in MUT than in WT animals. The primary role of dystrophin in skeletal muscle is to link the intracellular cytoskeleton to the extracellular matrix through a complex of transmembrane proteins called the dystrophin‐associated protein complex.^51^, ^52^ Desmin is thought to interact with this complex via α‐dystrobrevin and syncoilin, the latter that has been proposed to attach desmin to the complex at the sarcolemma to help organize myofibril structure.^45^ The fact that dystrophin levels are increased in MUT animals may hint at a compensatory effect to balance the lack of functional desmin in an effort to maintain myofibril alignment and attachment. Further indicative of the struggle of MUT skeletal muscle to maintain structural integrity are elevated levels of proteins associated with muscle membrane injury and repair. Dysferlin levels were increased two‐fold in MUT compared with WT. Dysferlin is involved in wound healing of membranous microlesions in a calcium‐dependent manner and mutations in dysferlin cause limb girdle muscular dystrophy 2B.^53^, ^54^ In skeletal muscle with functional dysferlin, injury causes an increase in dysferlin protein levels similar to what we see in MUT rats.^53^, ^54^, ^55^, ^56^ Annexin A2 works in concert with dysferlin to sense membrane damage and facilitate repair.^57^ Annexin A2 levels were similarly increased (32%) in MUT compared with WT animals. Finally, we found a four‐fold increase in muscle LIM protein in MUT compared with WT animals. Muscle LIM protein is a cytoskeleton associated protein that has been implicated in a variety of functions in the contractile apparatus such as myofibril organization and mechanical stress sensing.^58^, ^59^, ^60^ Similar to desmin, muscle LIM protein appears to interact with proteins around the z‐disk such as α‐actinin and has been suggested to be involved in the regulation of desmin expression in response to stretch.^61^, ^62^, ^63^ Overall, these data support the histological findings of structural instability and an increased susceptibility to injury.

To determine the capacity of MUT skeletal muscle to cope with stress, we employed the synergist ablation model to overload the plantaris muscle. Following 14 days of overload, macroscopic changes such as muscle mass did not differ between MUT and WT animals as both increased the weight of the plantaris muscle to a similar extent. However, maximal force output was 26% lower in the overloaded plantaris of MUT animals compared with WT, and this effect was concomitant with impaired muscle fibre hypertrophy. WT animals showed a right shift in muscle fibre distribution and a 23% increase in mean fibre area, whereas the MUT mean fibre area increased only 6%. This indicates that the MUT muscle fibres are not able hypertrophy to the same degree as WT muscle. Interestingly, WT animals also showed a small but pronounced increase in fibres with a very small cross‐sectional area that is absent in overloaded MUT muscle. Together, these data indicate that MUT muscles have a decreased ability to grow and are unable to split or generate new smaller fibres in response to a supra‐physiological load. The fact that the MUT rats were still able to increase muscle weight to a similar extent despite only mild hypertrophy and the absence of hyperplasia may point towards other coping mechanisms with loading, such as changes in connective tissue or muscle length.^64^ For patients affected by an orthologous mutation, these data in conjunction with the results from above indicate that while an increase in muscle mass and cross‐sectional area is possible when loading desminopathic muscle, caution should be used when therapists make decisions on the degree of loading. While still potentially useful in the prevention or slowing of muscle atrophy, the decreased ability to cope with very high loads and a higher susceptibility to muscle damage warrants a particularly careful approach to resistance exercise in these patients. This is similar to what has been seen in retrospective cohort studies of patients affected by dysferlinopathy, where vigorous physical activity and exercise during a young age could be associated with an earlier onset of symptoms with aging.^65^ Strategies known to cause hypertrophy and improved strength in the absence of high external loads (such as blood flow restriction training) could be an interesting consideration in this scenario, but more human data are needed to confirm such hypotheses.

In conclusion, we created a new animal model of desminopathy that is orthologous to the human mutation *DES* R350P. While models for this mutation are available in mice, we engineered the first CRISPR‐Cas9 rat model for the disease in an attempt to improve the biological resemblance of the human phenotype. As in the human disease, young individuals (120–150 days equivalent to 15–25 years old) show no overt muscle phenotype at an age corresponding to a pre‐clinical stage in human patients. Even though no overt phenotype was observed, microscopic evidence, characterized by regenerating fibres and protein aggregates, were already present in MUT rats. On a molecular level, desmin mutants showed an array of differentially regulated proteins that are associated with the structural integrity as well as injury of muscle fibres. Most of the levels of these proteins were increased in desmin mutant animals, likely as a compensatory response to dysfunctional intermediate filament alignment as a result of mutant desmin. Finally, a load challenge exacerbated the histological phenotype in the MUT animals resulting in impaired muscle fibre growth.

There is a wealth of intriguing avenues for future analysis of this animal model, most importantly concerning respiratory and cardiac muscle. As cardiac myopathies are a frequent cause of death in humans with a R350P mutation in desmin, investigating the heart will be pivotal to our understanding of the pathogenesis of the disease and attempts to improve life expectancy. In addition, future studies are necessary to elucidate metabolic complications induced by possible mitochondrial alterations in our model. We would be delighted to share this new model with other laboratories in an effort to find therapeutic solutions to ameliorate or delay the onset of symptoms associated with desminopathy.

## Author Contributions

H.T.L., A.A.M., H.Z.F., and K.B. conceived the study design. H.T.L., A.A.M., B.J.W., K.N.G., J.A.W., K.C.K.L., H.Z.F., and K.B. performed the experiments, H.T.L., A.A.M., H.Z.F., and K.B. analysed and interpreted results. All authors wrote, revised, and approved the final version of the manuscript.

## Conflict of Interest

The current study was funded by a generous gift from the Bertin‐Barbe Family to H.Z.F., and K.B. H.T.L., A.A.M., B.J.W., K.N.G., J.A.W., K.C.K.L., and H.Z.F. declare that they have no conflict of interest. K.B. is paid by professional sports and Olympic teams to consult on tendon/performance issues and has received grants, honoraria, and travel support from PepsiCo for nutrition research.

## Ethics Declarations

The authors of this manuscript certify that they comply with the ethical guidelines for authorship and publishing in the Journal of Cachexia, Sarcopenia and Muscle.^66^
